# High-throughput quantification of carboxymethyl lysine in serum and plasma using high-resolution accurate mass Orbitrap mass spectrometry

**DOI:** 10.1177/0004563219830432

**Published:** 2019-03-04

**Authors:** Naomi J Rankin, Karl Burgess, Stefan Weidt, Goya Wannamethee, Naveed Sattar, Paul Welsh

**Affiliations:** 1 Institute of Cardiovascular and Medical Sciences (ICAMS), BHF Glasgow Cardiovascular Research Centre, University of Glasgow, Glasgow, UK; 2 Glasgow Polyomics, University of Glasgow, Glasgow, UK; 3 Institute of Quantitive Biology, Biochemistry and Biotechnology, University of Edinburgh, Edinburgh, UK; 4 Primary Care and Population Health, Royal Free Campus, University College London Medical School, London, UK

**Keywords:** Carboxymethyl lysine, high-resolution accurate mass Orbitrap mass spectrometry, Orbitrap, isotope dilution mass spectrometry

## Abstract

**Background:**

Carboxymethyl lysine is an advanced glycation end product of interest as a potential biomarker of cardiovascular and other diseases. Available methods involve ELISA, with potential interference, or isotope dilution mass spectrometry (IDMS), with low-throughput sample preparation.

**Methods:**

A high-throughput sample preparation method based on 96-well plates was developed. Protein-bound carboxymethyl lysine and lysine were quantified by IDMS using reversed phase chromatography coupled to a high-resolution accurate mass Orbitrap Exactive mass spectrometer. The carboxymethyl lysine concentration (normalized to lysine concentration) was measured in 1714 plasma samples from the British Regional Heart Study (BRHS).

**Results:**

For carboxymethyl lysine, the lower limit of quantification (LLOQ) was estimated at 0.16 μM and the assay was linear between 0.25 and 10 μM. For lysine, the LLOQ was estimated at 3.79 mM, and the assay was linear between 2.5 and 100 mM. The intra-assay coefficient of variation was 17.2% for carboxymethyl lysine, 9.3% for lysine and 10.5% for normalized carboxymethyl lysine. The inter-assay coefficient of variation was 18.1% for carboxymethyl lysine, 14.8 for lysine and 16.2% for normalized carboxymethyl lysine. The median and inter-quartile range of all study samples in each batch were monitored. A mean carboxymethyl lysine concentration of 2.7 μM (IQR 2.0–3.2 μM, range 0.2–17.4 μM) and a mean normalized carboxymethyl lysine concentration of 69 μM/M lysine (IQR 54–76 μM/M, range 19–453 μM/M) were measured in the BRHS.

**Conclusion:**

This high-throughput sample preparation method makes it possible to analyse large cohorts required to determine the potential of carboxymethyl lysine as a biomarker.

## Introduction

Carboxymethyl lysine (CML) is an advanced glycation end product (AGE), produced *in vivo*, particularly under hyperglycaemic conditions, and available from the diet.^1,2^ Increased CML concentrations are associated with cardiovascular disease, diabetic nephropathy and retinopathy, chronic kidney disease and others.^2–4^ CML has been proposed as a potential biomarker of cardiovascular disease; however, conflicting results have been found.^4–6^ Enzyme linked immunosorbent assays (ELISAs) are available but suffer from steric hindrance of the antigen and interference from endogenous anti-AGE antibodies.^1,2^ Isotope dilution mass spectrometry (IDMS) methods are available for quantification of protein-bound CML, and samples require chemical reduction, protein denaturation, hydrolysis and drying prior to IDMS analysis. Published sample preparation methods are individual tube-based, which have limited throughput.^1,5^ Therefore, we developed a high-throughput sample preparation method using 96-well plates.

Published IDMS methods for CML quantification rely on multiple reaction monitoring (MRM) detection using triple quadrupole mass spectrometers (MS).^7^ In MRM, the first quadrupole is optimized to select for the parent ion of interest (CML) based on the mass to charge ratio (*m*/*z*). A collision cell fragments the parent ions into product ions,^7,8^ while the third quadrupole is optimized to select for specific product ions: the quantifier (for quantification) and the qualifier (for verification the identity).^7–9^ As triple quadrupole detectors provide excellent sensitivity and specificity, even in complex biofluids, they are widely used in clinical chemistry laboratories (e.g. toxicology, endocrinology and new born screening).^7–9^

There is increasing interest in the use of high-resolution accurate mass (HRAM) MS for absolute quantification of ions, including for routine clinical analysis and clinical research.^10,11^ HRAM MS relies on superior mass accuracy (typically sub 3 ppm), which allows excellent ion selectivity, provided appropriate mass-extraction windows (based on the theoretical *m*/*z* of the ion of interest) are chosen.^10–13^ HRAM MS analysis is commonly run in full scan mode, enabling the detection of all ionized compounds, without the need to optimize quadrupoles and collision energies for individual ions.^12^ The quantitative performance of HRAM MS now equals that of triple quadrupole mass spectrometry: in terms of sensitivity, mass accuracy, selectivity, although this does depend on the conditions, parameters and the metabolite of interest used.^10–12,14^ Some of the major advantages of HRAM MS are that data can be reanalysed retrospectively to investigate further biomarkers and that those biomarkers can be more easily identified (based on molecular formula).^10–12,14^ HRAM MS has been successfully used to quantify a number of small molecule groups: over 50 metabolites (including amino acids);^14^ amino acids (within 3 min);^15^ drugs and drug metabolites;^16^ circulating steroids^11^ and plasma metanephrines.^11^

We therefore optimized an HRAM IDMS method to quantify protein-bound CML and lysine and their deuterated internal standards using an Orbitrap Exactive mass spectrometer. To account for variation in plasma total protein concentration and variation introduced during sample preparation, the CML concentration was normalized to the lysine concentration.^5,6^ The three measures reported are CML (μM), lysine (μM) and normalized CML (μM per M lysine).

## Methods

### Sample preparation

Serum collected from one healthy volunteer, stored at –80°C in multiple aliquots, was used as a quality control (QC) sample. The Glasgow University Ethics Committee provided ethical approval for collection of anonymized samples for QC (Project number 200140133). Unthawed fasting EDTA plasma samples (*n* = 1714) from the 30th year re-examination of the British Regional Heart Study (BRHS),^17,18^ stored at –80°C, were randomized to 21 batches. Approval for collection was obtained from the local research ethics committees of the 24 towns where participants were recruited. All participants provided written informed consent to participate in the study.^18^ The study is consistent with the World Medical Association Declaration of Helsinki. The BRHS is a prospective study which recruited 7735 men between 1978 and 1980 from 24 British towns. At the 30-year re-examination, samples were collected from 1722 men between 2010 and 2012, with the men then being aged 71 to 92 years.^17^

A high-throughput 96-well deep-well plate method of sample preparation based on previously published methods was developed.^1,4–6^ Sodium tetraborate (Sigma, Dorset, UK), sodium borohydride (Alfa Aesar, Lancashire, UK), trichloroacetic acid (Sigma, Dorset, UK), hydrochloric acid (Sigma, Dorset, UK) were used for sample preparation. Plasma samples were defrosted for 90 min and centrifuged at 20,000 × *g* for 5 min. Ten microlitres of plasma were added to 300 *μ*L sodium tetraborate (0.2 M)/borohydride (0.1 M) buffer in a 96-well deep-well polypropylene plate (Thermo Fisher Scientific, Hemel Hempstead, UK). The samples were chemically reduced overnight at 4°C to prevent further production of CML (or other advanced glycation end products) during subsequent hydrolysis.^1^ The protein was denatured in 20% trichloroacetic acid, and the pellet was washed in 20% trichloroacetic acid. The protein pellet was then hydrolysed at 110°C in 600 *μ*L 6 M hydrochloric acid for 24 h, using a ceramic bead-bath. After hydrolysis, the samples were dried to completion at 95°C (approximately 24 h). Immediately prior to analysis, the samples were spiked with 10 *μ*L of 20 *μ*M CML-d_4_ (Toronto Research Chemicals, Ontario, Canada, 98% pure) and 10 *μ*L of 150 mM universally ^13^C labelled L-lysine:2HCl) (Cambridge Isotope Laboratories Inc., MA, USA, 98% pure) as internal standards (ISs) and reconstituted in 270 *μ*L of 5 mM nonafluoropentanoic acid (NFPA) (Sigma, Dorset, UK) as an ion-pairing agent.

CML (Toronto Research Chemicals, Ontario, Canada, 96% pure) and lysine (Sigma, Dorset, UK) were used to prepare calibrator samples: made up in water and then mixed with IS and NFPA. A seven-point calibration curve (CML: 0, 0.25, 0.5, 1, 2, 5, 10 μM and lysine: 0, 2.5, 5, 10, 20, 50, 100 mM) was used to quantify both CML and lysine relative to their ISs. The concentration ranges were chosen based on the concentrations previously reported using IDMS quantification.^1,6^ Previous studies demonstrated that acid hydrolysis of calibration solutions did not alter peak area; therefore, calibrator samples were not hydrolysed.^1^

### Chromatography and HRAM mass spectrometry

Chromatography was carried out on an UltiMate 3000 RSLC system (Thermo Fisher Scientific, Hemel Hempstead, UK) using an ACQUITY UPLC BEH C18 column (100 mm × 2.1 mm, 1.7 μm column, Waters, Wilmslow) with VanGuard pre-column (Waters, Wilmslow, UK). Mobile phase A was 5 mM NFPA (Sigma, Dorset, UK) in HPLC grade water (Fisher, Loughborough, UK). Mobile phase B was HPLC grade acetonitrile (Fisher, Loughborough, UK). The column was maintained at 50°C, and samples were eluted with a linear gradient over 9.0 min at a flow rate of 0.3 ml/min. Starting conditions were 90% mobile phase A, decreasing to 20% between 0.1 to 4.6 min; this was held between 4.6 and 6.1 min, then increased to 90% at 6.2 min and held until 9.0 min to re-equilibrate the column. The injection volume was 5 *μ*L, and samples were maintained at 5°C prior to injection. For HRAM MS, an Orbitrap Exactive (Thermo Fisher Scientific, Hemel Hempstead, UK) was operated in high-resolution full scan positive mode, at a scan range of 120–250 *m*/*z*, a probe temperature of 150°C and capillary temperature 275°C. The mass resolution was 50,000, providing a mass accuracy of less than 1 ppm. A mass calibration was performed prior to each batch using Pierce LTQ Velos positive ion calibration solution (Thermo Fisher Scientific, Hemel Hempstead, UK). TraceFinder 3.3 (Thermo Fisher Scientific, Hemel Hempstead, UK) was used to automatically detect peaks within expected retention time and mass extraction windows.

Protein-bound CML and lysine concentrations were calculated based on integrated areas relative to those of the ISs. The normalized CML concentration (μM/M lysine) was calculated from the measured CML and lysine concentrations observed (CML/lysine × 1,000,000). This allows for variation in total protein concentration to be accounted for, analogous to reporting HbA1c in relation to haemoglobin concentration.^19^ It also allows variation in hydrolysis to be accounted for. Once the method was optimized, 1714 samples from the BRHS were analysed.

## Results and discussion

### Isolation and detection

Mean Pearson correlation coefficients, mean response factors (gradients) and mean y-intercepts for protein-bound CML and lysine calibration were calculated (Table 1).

**Table 1.** Figures demonstrating assay performance and median concentration observed in BRHS plasma samples.

	CML	Lys	CML (normalized)
Calibration			
Pearson correlation coefficient (r^2^) (SD)	0.9994 (0.003)	0.9993 (0.0008)	NA
Gradient (SD)	0.0223 (0.0102)	0.0016 (0.0007)	NA
Intercept (SD)	0.0020 (0.0008)	1.146 (1.18986)	NA
Water-based QC			
Measured concentration (μM)	0.29	2,377	NA
Inter-assay CV (%)	10.1	18.3	NA
Estimated LOB (μM)	0.13	136	NA
Estimated LOD (μM)	0.12	1250	NA
Estimated LOQ (μM)	0.16	3789	NA
Chosen LOQ	0.25	2500	NA
Serum based QC			
Intrasample CV	2.7	2.1	3.9
Intra-assay CV	17.2	9.3	10.5
Inter-assay CV	18.1	14.8	16.2
BHRS plasma samples			
Median measured Concentration (μM)	2.5	39,773.5	65
Interquartile range (μM)	2.0 to 3.2	36,109 to 43,210.6	54 to 76
Estimated reference range (μM)	1.1 to 5.6	26,182 to 57,677	34 to 123

CML: carboxymethyl lysine; LOQ: limit of quantification; LOB: limit of blank; LOD: limit of detection.

**Figure 1.**
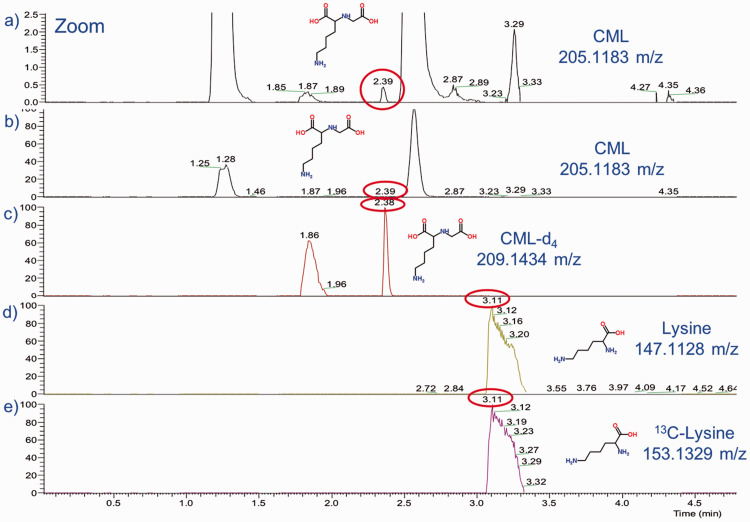
Extracted-ion-chromatograms (0–5 min): (a) Close-up of CML (and other metabolites) in serum with *m*/*z* of 205.1183 and retention time of 2.39 min (note splitting of the CML peak [at 1.87 and 2.39 min] and closely eluting peak thought to be valylserine or serylvaline; (b) CML (and other metabolites) in serum with *m*/*z* of 205.1183 and retention time of 2.39 min; (c) deuterated CML in serum with *m*/*z* of 209.1343 and retention time of 2.38 min (note splitting at 1.86 and 2.38 min); (d) lysine in serum with *m*/*z* of 147.1128 and retention time of 3.11 min; (e) universally labelled ^13^C-lysine in serum with *m*/*z* of 153.1329 and retention time of 3.11 min. CML: carboxymethyl lysine.

Separation of CML from the closely eluting peak, suspected to be valylserine or serylvaline, was good in most (1614 [97%]) samples (Figure 1). Valylserine and serylvaline are dipeptides composed of valine and serine with the same molecular formula (C_8_H_16_N_2_O_4_) and *m*/*z* as CML and are likely to be produced during acid hydrolysis. Since this is an isobaric interference, reduction of the mass extraction window cannot eliminate this interferent. The CML and CML-d4 appear to have isomerized, resulting in two peaks with the same *m*/*z* at two different retention times (1.86 and 2.38 min). The peak with the latter retention time was chosen for integration due to better peak shape.

### Limits of blank, detection and quantification

In IDMS, the lower limit of detection (LLOD) and quantification (LLOQ) are usually determined by calculating the signal to noise ratio (SNR) in a spectrum, with an SNR of 3 being used as an LLOD and an SNR of 10 (with accuracy of 80–120% and <20% imprecision) being used as an LLOQ.^8^ Due to the signal processing of the Orbitrap Exactive, with baseline removal inherent in Orbitrap data acquisition, there is generally no noise in the extracted ion chromatogram.^11^ Therefore, the SNR for all peaks was infinity, and SNR cannot be used to estimate LLOD and LLOQ. Instead, the LLOD and LLOQ were estimated based on the slope and SD of the y-intercept of the calibration curve.^20^

In the lowest water-based calibrator sample (0.25 μM), the mean CML concentration (over 21 batches) was 0.29 μM (<20% bias), with an inter-assay coefficient of variation (CV) of 10.1% (<20% variation). In the water-based calibrators, the results were linear between 0.25 μM and 10 μM. The limit of blank (LOB) for the zero-calibrator sample was estimated as 0.13 μM based on an average concentration of 0.04 μM and a standard deviation of 0.06 (LOB = mean + 1.645 SD).^21^ The LLOD was estimated as 0.12 μM, by multiplying the SD of the y-intercept by 3.3 and dividing by the slope of the calibration curve.^20^ The LLOQ was estimated as 0.16 μM, by multiplying the SD of the y-intercept by 10 and dividing by the slope of the calibration curve.^20^ The concentration of the lowest water-based calibrator (0.25 μM) was chosen as the LLOQ, in order to avoid extrapolation,^22^ since CML is endogenous to serum samples. Only one plasma sample was observed with a CML concentration below the LLOQ of 0.25 μM. Thirteen samples were observed with a CML concentration of over 10 μM (ranging between 11.9 and 17.4 μM). Since there was no evidence of detector saturation with the closely eluting isobaric contaminant peak present at approximately 1000 times higher concentrations (based on peak area), the linearity can be extrapolated.

In the lowest water-based calibrator sample, the mean lysine concentration (over 21 batches) was 2377 μM (<5% bias), with an inter-assay CV of 18.3% (<20%).^22^ In the water-based calibrators, the results were linear between 2500 μM and 100,000 μM. The LOB for the zero-calibrator sample was estimated as 136 μM based on an average concentration of –539 μM and an SD of 410 (LOB = mean + 1.645 SD).^21^ The LLOD was estimated as 1250 μM, by multiplying the SD of the y-intercept by 3.3 and dividing by the slope of the calibration curve.^20^ The LLOQ was also estimated as 3789 μM, by multiplying the SD of the y-intercept by 10 and dividing by the slope of the calibration curve.^20^ The concentration of the lowest water-based calibrator, 2500 μM, was chosen as the LLOQ, in order to avoid extrapolation,^22^ since lysine is endogenous to serum samples. No serum samples were observed with a lysine concentration below 2500 μM (all ≥ 8,343 μM). No serum samples were observed with a lysine concentration over 100,000 μM (all ≤ 58,231 μM).

### Serum-based QC

Six serum QC samples were prepared and re-injected 16 times each. The mean intra-assay, intra-sample CVs were 2.7% for CML, 2.1 for lysine and 3.9% for normalized CML. This demonstrates that repeated analysis of the same sample preparation is robust. The CVs for normalized CML are increased, since the variability of both the CML measurement and the lysine measurement is contributing to the overall variability.

The intra-assay CV (based on 30 freshly prepared and reconstituted samples, each injected only once) was 17.2% for CML, 9.3% for lysine and 10.5% for CML normalized to lysine. The CV for normalized CML is lower than that of directly measured CML in serum samples, as normalization accounts for variation incorporated during individual sample preparation.

The overall inter-assay CV was 18.1% for CML, 14.8% for lysine and 16.2% for CML: lysine. The inter-assay CVs for CML (directly measured and normalized) are outside the target CV of 15% recommended by Food and Drug Administration and other guidelines for validation of bioanalytical methods within regulated environments.^22^ In non-regulatory environments, a CV of 20–25% is a commonly used target.^23^ The variation may have been introduced during high-throughput sample preparation, particularly during hydrolysis or during HRAM MS analysis.

It is recommended that the normalized CML concentration is used for clinical research studies, as it accounts for variation in sample preparation and in blood total protein concentrations. This is analogous to reporting HbA1C in relation to haemoglobin concentration.^19^

**Figure 2.**
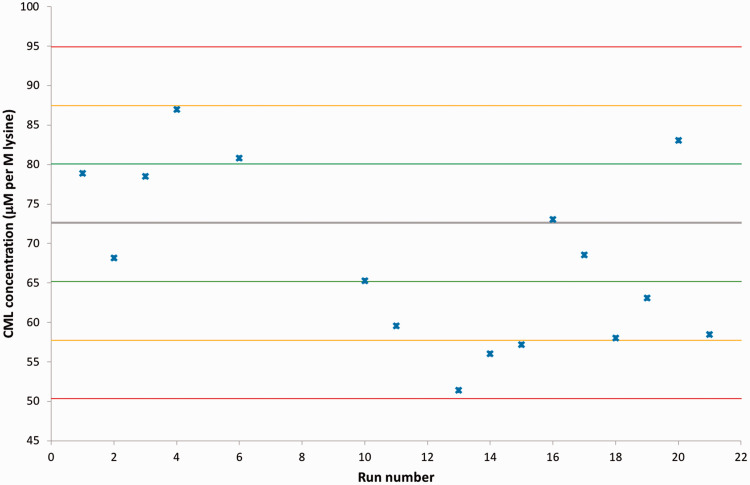
Levy-Jennings plot displaying the variability of the measured concentration of CML (normalized to the measured lysine concentration) in the quality control serum samples run with every batch. The mean normalized CML concentration derived from previous analysis of 12 quality control serum samples is referenced as the grey line. The green, yellow and red lines reference the mean ± 1 2 and 3 SD, respectively. Note for some runs co-elution with an isobaric interferant or contamination of the QC sample meant that CML concentration could not be measured. CML: carboxymethyl lysine.

To assess the variability of the HRAM MS analysis and the sample preparation, a serum-based QC was prepared and run with every plate. The normalized CML concentration obtained was within QC limits, according to the Westguard Multi-rules, for all but one batch, and all but three within 2 SD of the mean of 12 run-in samples (Figure 2). Unfortunately, in some runs, this serum QC sample was contaminated – either from an unknown contaminant eluting over a wide retention range or from co-elution of the isobaric peak (suspected to be valylserine or serylvaline) (Figure 1).

### Batch-to-batch variation

**Figure 3.**
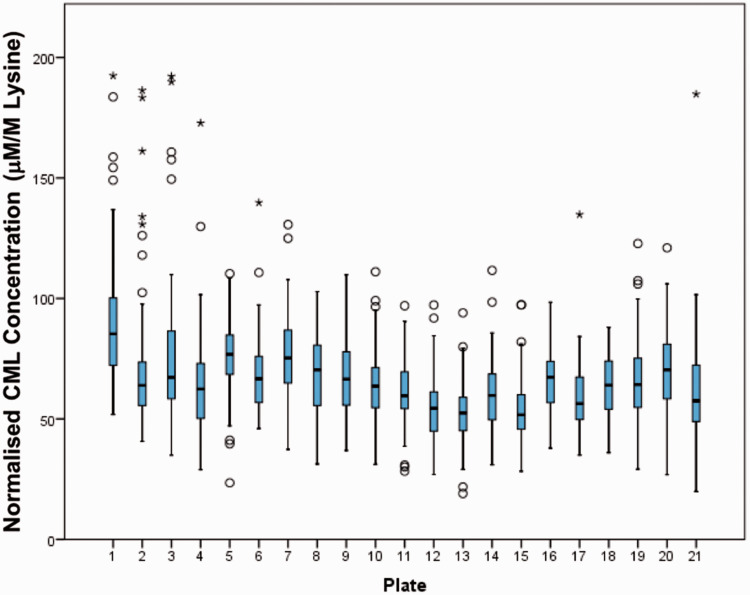
Box plots showing median (line), box (interquartile range) and whiskers (<1.5 × IQR) for normalized CML concentration in BRHS plasma samples run in each batch, arranged by plate number (*n* = 95 per batch). The circles represent outliers (<3 × IQR) and the stars represent extreme outliers (>3 × IQR). No obvious trends are observed from batch to batch; samples were randomized before sample preparation. Normalized CML concentrations of ≥ 200 mM/M lysine were excluded from the figure for clarity. CML: carboxymethyl lysine.

Since no external or commercial QC material exists, the overall mean, median and interquartile range (IQR) was monitored for each batch of 95 samples to check for batch effects. The batch-to-batch values are expected to vary, since each batch includes different samples; however, no obvious trends were observed in normalized lysine concentration (Figure 3).

To attempt to investigate the stability of the dried hydrolysed samples, 12 samples which were stored for three months at –80°C were reconstituted with NFPA and analysed by HRAM MS. The mean CML, lysine and normalized CML concentrations were not significantly different. To check for unwanted trends in the data over time, the box plots were arranged by date run on the LC-MS, by date of sample preparation and by delay between sample preparation and LCMS run. No obvious trends were observed (data not shown).

### EDTA plasma versus serum samples

Paired samples of serum (serum separator vacutainers) or plasma (K^+^EDTA vacutainers) from seven healthy volunteers were run in triplicate (Table 2). The concentrations observed were not statistically significantly different for all three analytes. CML concentrations were also found to be similar in plasma versus serum samples in a previous study.^4^

**Table 2.** Comparison of results from paired serum and EDTA plasma samples (seven paired samples run in triplicate). Results of paired t-test demonstrated no significant (ns) difference between the two sample types.

	CML (μM)	Lys (μM)	CML (μM/M lys)
Serum (*n* = 7) (mean [SD])	2.543 (0.44)	44,881 (3646)	57 (8.0)
EDTA plasma (*n* = 7) (mean [SD])	2.509 (0.37)	44,675 (2506)	56 (6.5)
*P* (paired *t*-test)	0.665 (ns)	0.740 (ns)	0.697 (ns)

EDTA: ethylenediaminetetraacetic acid; CML: carboxymethyl lysine.

### Throughput

We developed a 96-well deep-well method of sample preparation based on previously published methods individual tube methods.^1^ The hands-on preparation time (not including incubations) is reduced more than five-fold compared with the estimated sample preparation time reported for individual tube methods^1^ (Table 3). It also uses lower volumes of reagents. MS analysis time is slightly increased: 9-min run compared with the 7.5-min run published.^4^ However, analysis of a 96-well plate can still be completed within an overnight run.

**Table 3.** Comparison of hands-on sample preparation time using the 96-well versus individual tube method and comparison of HRAM MS analysis time for 1000 samples.

Per 1000 samples	Traditional method (hr)	HT method (h)
Sample preparation (hands-on)	150	26
Chromatography and detection	125	150

### Comparison with other IDMS methods

**Figure 4.**
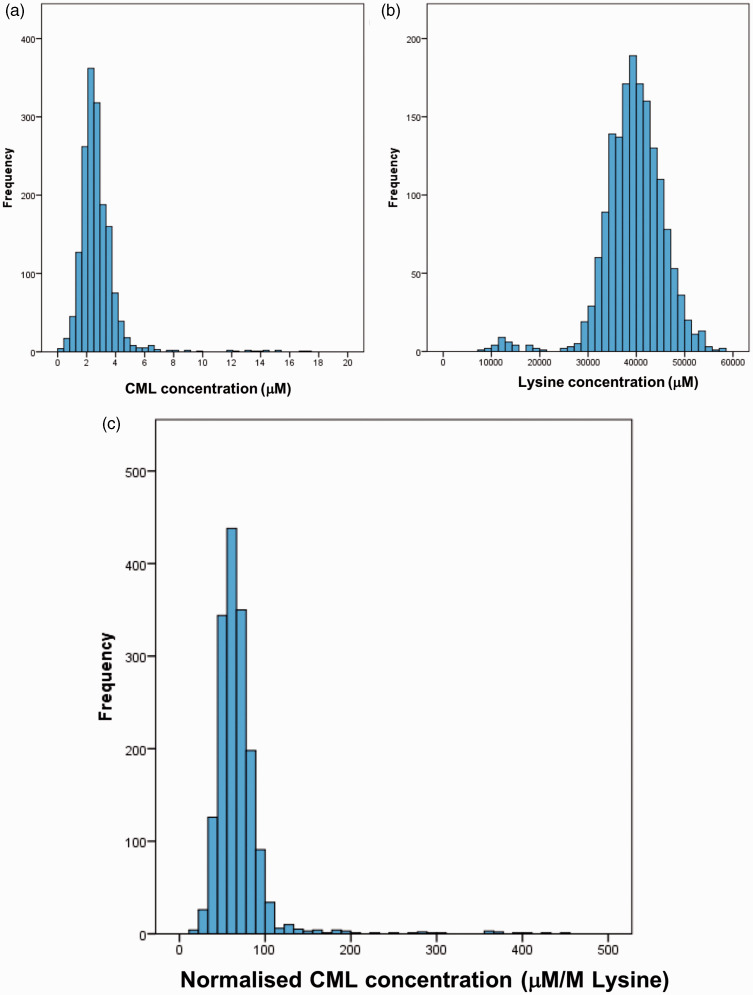
Histogram of (a) CML concentration (μM); (b) lysine concentration (μM); (c) normalized CML concentration (μM/M lysine) in 1664 BRHS EDTA plasma samples. CML: carboxymethyl lysine.

The CML concentration was measurable in 1664 samples from the BRHS. For 50 samples (3%), the CML peak co-eluted with the isobaric peak (suspected to be valylserine or serylvaline) or there was broad contamination. The CML and normalized CML concentrations were positively skewed and results for lysine were negatively skewed (Figure 4).

A mean of 2.7 μM (full range: 0.2–17.4 μM) with an SD of 1.4 μM and a median of 2.5 μM (IQR: 2.0–3.2 μM) was found for CML concentration in the BHRS samples (*n* = 1664). A reference range of 1.1 to 5.6 μM was calculated (mean ± 1.96 SD, after log transformation). It should be noted that the samples obtained at the 30th year re-examination of the BRHS were from white European males aged 71 to 92 years and that up to 444 of the participants had a diagnosis of CVD.^17^

The mean CML concentration of 2.7 μM measured is similar to the mean of 2.8 μM (SD 0.4 μM), previously reported for 10 healthy controls^1^ (Table 4). The BRHS median CML concentration of 2.5 μM is also similar to the median concentration of 2.9 μM (range 1.7 to 4.4 μM) observed in 31 individuals with type 1 diabetes mellitus with normal renal function.^5^

**Table 4.** Comparison of CML and normalized CML concentration observed by HRAM MS with high-throughput sample preparation vs. LC-MS/MS with individual tube-based sample preparation.

		Mean (±SD) or median (IQR) in health		Mean (±SD) or median (IQR) in disease	
Reference	Sample type	CML (μM)	Population information	CML (μM)	Population information
This study	Fasting EDTA plasma	2.7 (±1.4)2.5 (2.0 to 3.2)	1664 Caucasian Europeans aged 71–92, 444 with CVD	NA	NA
Gaens et al.^24^	EDTA plasma and serum	1.61 (±0.38)	738 Individuals in the Dutch Hoorn Study	1.77 (±0.45)	532 Individuals in the Dutch CODAM study
Teerlink et al.^1^	Plasma	2.8 (±0.4)	10 Healthy individuals	7.26 (±1.36); 8.01 (±3.8)	17 Individuals on haemodialysis; 9 individuals on peritoneal dialysis
Liew-A-Fa et al.^5^	Plasma	2.9 (1.7 to 4.4)	31 Individuals with T1DM and normal eGFR (>80 mL/min)	4.9 (2.0 to 12.6)	29 Individuals with T1DM and decreased eGFR (<80 mL/min)
		Normalized CML (μM/M lysine)		Normalized CML (μM/M lysine)	
This study	Fasting EDTA plasma	69 (±34)65 (54 to 76)	1664 Caucasian Europeans aged 71–92, 444 with CVD	NA	NA
Anwar et al.^25^	Fasting EDTA	158 (±26)	21 Healthy children	190 (±38)	27 Children with autism spectrum disorder
Maessen et al.^26^	Serum	68 (56–76)	18 Sedentary individuals	80 (73 to 89)	18 Athletes
De Courten et al.^27^	Serum	NA	NA	77.6 (±14)	20 Overweight or obese individuals
Linssen et al.^28^	Serum	31.6 (27.4 to 37.3)	>200 Individuals in top tertile for diastolic function	33.6 (28.5 to 38.7)	>200 Individuals in bottom tertile for diastolic function
Hanssen et al.^29^	Plasma	52.1 (46.1 to 59.6)	∼70 Individuals in bottom tertile for AGE score	80.4 (72.1 to 91.7)	∼70 Individuals in top tertile for AGE score
Gopal et al.^30^	EDTA plasma	82.9 (±19.3)	44 Ex-smokers	61.6 (±15.6)	88 Individuals with COPD
Hanssen et al.^6^	Fasting EDTA plasma	34 (29 to 39)	733 Individuals without prior CVD	33 (27 to 38)	558 Individuals with prior CVD
Hull et al.^4^	Fasting serum and plasma	Medians 132 to 140	Single pool of 10 healthy individuals measured with different sample processing	NA	NA
Ga Van Eupen et al.^31^	Fasting EDTA plasma	92.5 (±15.7)	169 Individuals without Diabetes	104.6 (±19.4)	165 Individuals with T1DM
Rabbani et al.^32^	Plasma	NA	NA	52 (±14)	52 Individuals with T2DM and microalbuminuria
Engelen et al.^33^	Plasma	NA	NA	49.9 (±11.9)	125 Individuals with T2DM and microalbuminuria
Thornalley et al.^34^	Plasma	21 (±5)	Five healthy controls	NA	NA

AGE: Advanced glycation end-product; COPD: chronic obstructive pulmonary disease; CVD: cardiovascular disease; EDTA: ethylenediaminetetraacetic acid; eGFR: estimated glomerular filtration rate; IQR: interquartile range; NA: not applicable; SD: standard deviation; T1DM: type 1 diabetes mellitus; T2DM: type 2 diabetes mellitus; CML: carboxymethyl lysine.

The mean lysine concentration in the BHRS samples was 39,490.5 μM, with an SD of 6268.6 μM, after hydrolysis. The median was 39,773.5 μM (IQR: 36,109.0–43,210.6). A reference range of 26,182 to 57,677 μM was calculated (mean ± 1.96 SD, after log transformation). To our knowledge, no reference ranges have been reported for lysine concentration in serum or plasma hydrolysate, as this is not a physiologically relevant measure. The lysine concentration correlated with the albumin concentration (r^2^ = 0.24), demonstrating that variation in protein concentration and variation in sample preparation contribute to the variation in lysine concentration.

A mean normalized CML concentration of 69 μM/M lysine (range: 19–453; SD 34) was reported in the BRHS. The median was 65 μM/M lysine (IQR: 54–76). A reference range of 34 to 123 μM/M was estimated from the log-transformed data (mean ± 1.96 SD).

The median normalized CML concentration of 65 μM/M lysine measured is similar to the median of 68 μM/M lysine, previously reported for 18 sedentary individuals^26^ (Table 4). It is also broadly similar to the medians of 51 μM/M lysine and 80 μM/M lysine reported for 70 individuals in the top and bottom tertile of AGE score, respectively.^29^

The mean of 69 μM/M lysine observed in the BRHS samples was far lower than the medians reported by Hull et al.: 132 to 140 μM/M lysine^4^ (Table 4). They measured lysine concentration using IDMS; however, they did not report the raw CML or lysine concentrations observed. Their range is based on repeated analysis of a pooled sample from 10 healthy volunteers analysed under different preanalytical conditions, none of which were found to significantly affect the CML concentration.^4^ It is also far lower than the mean of 158 μM/M lysine reported in 21 healthy children^25^ and lower than the mean of 83 μM/M lysine reported in 44 ex-smokers^30^ and of 93 μM/M lysine reported in 169 individuals without diabetes.^31^

The median normalized CML concentration is about double that reported by Hanssen et al.^6^: 34 μM/M lysine (IQR: 29 to 39) based on 558 individuals without prior CVD. They derivatized CML with 1-butanol: HCl, as an alternative to using NFPA as an ion-pairing agent, before IDMS analysis. The sample was split after hydrolysis and the lysine concentration was measured separately, again using IDMS. Hanssen et al. did not report the raw CML or lysine concentrations observed. Perhaps incomplete derivatization or differences in sample population account for the difference in ranges observed between our study and theirs. It is also approximately double that reported for over 200 individuals in the top tertile for diastolic function^28^ and more than double that reported in five healthy individuals.^34^

## Conclusion

CML is a challenging AGE to measure, as it is being detected in the presence of other amino acids (including lysine) present at 1000 times higher concentrations in the sample. However, the results suggest that this is a robust method for the quantification of CML (normalized to lysine), despite reducing the hands-on time (and reagent volumes used) for sample preparation substantially. There are no gold standard methods available for comparison at present, only ELISAs and other IDMS methods. Our method appears to compare relatively well to other IDMS-based methods. At present, we recommend this method for research use only. Further work is required in clinical research to determine whether CML is indeed a useful biomarker. The increased throughput provided by this sample preparation method will aid this endeavour. If CML is a useful biomarker, then it will be appropriate to evaluate whether the improved mass accuracy of an ultra high-resolution instrument is better for sensitivity and selectivity than a triple quadrupole instrument for this analyte.
